# Evidence-based prescribing of opioids after laparotomy: A quality-improvement initiative in gynecologic oncology

**DOI:** 10.1016/j.gore.2024.101396

**Published:** 2024-04-27

**Authors:** Allison H. Kay, Rachel Levy, Nancy Hills, Allyson Jang, Alison Mcgough-Maduena, Natalia Dematteo, Melissa Mark, Stefanie Ueda, Lee-may Chen, Jocelyn S. Chapman

**Affiliations:** a Division of Gynecologic Oncology, Department of Obstetrics, Gynecology & Reproductive Sciences, University of California San Francisco, 490 Illinois Street, 10th Floor, Box 0132, San Francisco, CA 94143, USA; b Department of Epidemiology and Biostatistics, University of California San Francisco, 550 16^th^ Street, 2nd Floor, San Francisco, CA 94158, USA

**Keywords:** Postoperative pain, Opioids, Perioperative care

## Abstract

**Introduction:**

Across specialties, surgeons over-prescribe opioids to patients after surgery. We aimed to develop and implement an evidence-based calculator to inform post-discharge opioid prescription size for gynecologic oncology patients after laparotomy.

**Methods:**

In 2021, open surgical gynecologic oncology patients were called 2–4 weeks after surgery to ask about their home opioid use. This data was used to develop a calculator for post-discharge opioid prescription size using two factors: 1) age of the patient, 2) oral morphine equivalents (OME) used by patients the day before hospital discharge. The calculator was implemented on the inpatient service from 8/21/22 and patients were contacted 2–4 weeks after surgery to again assess their opioid use at home.

**Results:**

Data from 95 surveys were used to develop the opioid prescription size calculator and are compared to 95 post-intervention surveys. There was no difference pre- to post-intervention in demographic data, surgical procedure, or immediate postoperative recovery. The median opioid prescription size decreased from 150 to 37.5 OME (p < 0.01) and self-reported use of opioids at home decreased from 22.5 to 7.5 OME (p = 0.05). The refill rate did not differ (12.6 % pre- and 11.6 % post-intervention, p = 0.82). The surplus of opioids our patients reported having at home decreased from 1264 doses of 5 mg oxycodone tabs in the pre-intervention cohort, to 490 doses in the post-intervention cohort, a 61 % reduction.

**Conclusions:**

An evidence-based approach for prescribing opioids to patients after laparotomy decreased the surplus of opioids we introduced into our patients’ communities without impacting refill rates.

## Highlights

• We developed an easy-to-use evidence-based calculator for post-laparotomy opioid prescription sizes.
• Use of this calculator decreased our median opioid prescription size by 75% and amount of leftover, unused opioids by 61%
• The median amount of 5 mg oxycodone used by our post-intervention patients after discharge to home was one pill.

## 1 Introduction

Across specialties, surgeons over-prescribe opioids to patients after surgery (Ladha et al., 2019, Thiels et al., 2017, Thiels et al., 2018). While opioid prescription rates have decreased nationally since 2012, several studies demonstrate that surgeons continue to overestimate patients’ needs for postoperative pain medication (As-Sanie et al., 2017, Centers for Disease Control and Prevention, National Center for Injury Prevention and Control, 2023, Griffith et al., 2018, Hill et al., 2017). Within our gynecologic oncology practice at a single academic institution, we have reduced our median post-discharge opioid prescription for open surgeries from the equivalent of 90 doses of 5 mg oxycodone per patient in 2012 to 20 doses of 5 mg oxycodone in 2021, but subsequent post-discharge surveys confirm that our patients still need fewer opioids than prescribed (Kay et al., 2023).

While on an individual basis, over-prescription may improve patient satisfaction with their pain management and decrease requests for refills, collectively it leads to a surplus of opioids that negatively impacts our patients and their communities. Repeated studies show that patients do not dispose of their leftover, unused opioids and there is a risk for diversion of the medication by a patient or their family and friends (Bartels et al., 2016, Bates et al., 2011, Hasak et al., 2018, Rachel N. Lipari, 2017). Larger opioid prescriptions confer an increased risk for developing persistent opioid use (Chan et al., 2021, Wright et al., 2019). Additionally, larger opioid prescriptions are associated with risk of opioid overdose in a family member (who was not prescribed opioids) (Khan et al., 2019). As a surgical community, we should care about every excess opioid prescribed to our patients. Our postoperative gynecologic oncology patient survey data in 2021 identified an excess of 1331 doses of 5 mg oxycodone prescribed per 100 patients (Kay et al., 2023).

A major barrier to prescribing opioids with accuracy at the time of postoperative hospital discharge is the lack of evidence-based tools available to guide prescription size. While some groups have proposed set ranges of opioids for patients after specific procedures (e.g. 0–20 pills after hysterectomy), uniform prescribing guidelines will inevitably fail to meet the needs of some patients (Boitano et al., 2020, Michigan Opioid Prescribing Engagement Network, 2020, Overton et al., 2018). Furthermore, without evidence-based tools utilizing objective patient data, there is a risk of implicit bias impacting opioid prescriptions leading to inequity in care (Herb et al., 2021).

We sought to develop a personalized postoperative opioid prescription size calculator that would use readily available objective patient data and reduce the risk of over-prescribing without increasing the rate of refill. After development, we implemented our calculator on our gynecologic oncology inpatient service for postoperative patients discharging home after open surgery.

## 2 Methods

Our project was identified as a quality improvement project by WCG IRB, with whom our university’s IRB has a reliance agreement. We conducted this quality improvement project on the inpatient gynecologic oncology service at a single, university academic institution with both obstetric & gynecology residency and gynecologic oncology fellowship training programs. We targeted patients after open surgery alone as our typical prescription for patients after minimally invasive surgery is already minimal (0–5 doses of 5 mg oxycodone). All patients who undergo laparotomy receive an epidural or a transversus abdominis plane (TAP) block and oral analgesics such as acetaminophen, ibuprofen, and gabapentin for postoperative pain control. Our patients are from socio-economically diverse backgrounds and from both rural and urban settings across northern California. Approximately three patients a week undergo open surgery, and they are cared for postoperatively by a resident team, two nurse practitioners, and an on-service gynecologic oncology fellow. Any of these providers may write the discharge opioid prescriptions. New residents rotate onto the gynecologic oncology inpatient service every five weeks, with all levels of obstetrics & gynecology residents represented. At the outset of this project, the median prescription size at the time of hospital discharge was the equivalent of 20 doses of 5 mg oxycodone.

We conducted telephone or virtual visit surveys of 95 patients 2–4 weeks after surgery. We chose this timeline as it is our experience that nearly all patients report they are no longer taking pain medication at their 2-week postoperative visit. We included adult patients who had undergone an open surgery (including conversion cases) by a gynecologic oncologist, were cared for primarily by our inpatient gynecologic oncology team and were discharged to home. We excluded patients who were readmitted prior to the survey. After obtaining verbal consent to participate, we conducted our four-question survey.

1. Did you have any opioid pain medication that you did not use/need? If so, how much?
2. The number of opioid pills you were given was: a) too few, b) just right, or c) too many?
3. Did you need a refill of the opioid medication prescribed? If so, was asking for a refill a) easy or b) challenging?
4. Approximately a month prior to surgery, were you using a prescription opioid pain medication on a daily basis?

We abstracted patient and perioperative data from the electronic medical records (EMR) of survey respondents. We used paired chart review and survey responses to develop our model of self-reported opioid use. Of the 95 patients surveyed (i.e. our “pre-intervention” cohort), 9 were identified as outliers using more than 40 doses of 5 mg oxycodone (or equivalent opioid). These 9 patients were excluded from the development of our model. Compared to the patients who used ≤ 40 doses of oxycodone, these patients were more likely to have a diagnosis of anxiety, diagnosis of chronic pain or opioid tolerance as defined by the Food and Drug Administration (FDA), and have used > 37.5 OME the day before hospital discharge.

Using data from our surveys, we built a model to predict self-reported opioid use (expressed as a number of 5 mg oxycodone doses). We used univariate negative binomial models to calculate association coefficients for a range of individual patient and perioperative variables. We examined different multivariable negative binomial models using variables that were significant at the 0.05 level in univariate analysis. Nested models were compared using likelihood ratio tests. Based on these tests, our final model included two variables: age divided into three categories (18–29, 30–59, 60 + years) and oral morphine equivalents (OME) used the day before hospital discharge in four categories (0–7.5, 7.6–22.5, 22.6–37.5, >37.5 OME). We used the model predictions to derive estimates for each of the 12 possible patient categories (combining age category and OME category). We used standard ratios of oral and intravenous medication to oral morphine to calculate OME (Table 1) (Naidu and Schumacher, 2015).

**Table 1:** Conversion of common opioids to oral morphine equivalents (OME).

Opioid	Equivalent OME
PO Hydrocodone 1 mg	1
PO Oxycodone 1 mg	1.5
PO Hydromorphone 1 mg	4
PO Tramadol 1 mg	0.25
PO Codeine 1 mg	0.15
IV Hydromorphone 1 mg	20
IV Fentanyl 1mcg	0.3

Once we developed our final model for self-reported postoperative opioid use, we used it to develop a clinical calculator to suggest a discharge prescription size. We examined how increasing the model estimates by 30 %, 50 %, 100 %, 150 % and 200 % would change the proportion of patients who would have been under-prescribed (i.e. would need a refill). The refill rate of our pre-intervention cohort was 12.6 %. We found that an increase of 50 % over the predicted estimates minimized the surplus pills prescribed when compared to actual usage and had a predicted under-prescription, or refill rate, of 15 % which our division felt was an acceptable outcome. Our final calculator is shown in Table 2. Providers were recommended to prescribe no more than a maximum of 40 doses of an opioid.

**Table 2:** Calculator for recommended post-discharge opioid prescription sizes for gynecologic oncology patients after open surgeries.

		**Recommended prescription size**			
**Age**	**OME used the day before hospital discharge**	**5 mg oxycodone tabs**	**2 mg hydromorphone tabs**	**50 mg tramadol tabs**	**5 mg hydrocodone tabs**
18–29 years	0–7.5	1	1	1	2
18–29 years	7.6–22.5	2	2	2	3
18–29 years	22.6–37.5	3	3	2	5
18–29 years	>37.5	4	4	3	6
30–59 years	0–7.5	2	2	2	3
30–59 years	7.6–22.5	13	13	8	20
30–59 years	22.6–37.5	22	21	14	33
30–59 years	>37.5	33	31	20	50
60 + years	0–7.5	2	2	2	3
60 + years	7.6–22.5	8	8	5	12
60 + years	22.6–37.5	13	13	8	20
60 + years	>37.5	20	19	12	30

We introduced our calculator on the inpatient gynecologic oncology service August 21, 2022 and gave an introductory presentation at the beginning of each block of residents about the goals of the project and how to use our calculator. We emphasized that the calculator makes a recommendation, and that patient and provider could decide on a smaller or larger prescription size as appropriate.

After we launched the use of our calculator on the inpatient service, we contacted another 95 postoperative patients discharged to home after open surgeries 2–4 weeks later. We used the same four question survey and simultaneously conducted chart review from their medical records. These patients formed our post-intervention cohort. These post-intervention surveys were conducted over eight months.

For our statistical analysis, we compared patient and perioperative characteristics between the pre-intervention and post-intervention cohorts. We also compared survey responses. Our primary aim was the reduction of leftover, unused opioids that our patients have after they no longer need opioids for pain. We compared categorical variables using chi-square tests. We used medians to represent continuous variables and compared them using Wilcoxon rank-sum tests.

This manuscript was written using SQUIRE 2.0 guidelines.

## 3 Results

We implemented the calculator on our inpatient service on August 21, 2022 without modification. We performed an initial analysis of our data in November 2022 for our first plan-do-study-act (PDSA) cycle and realized that we were unable to say with certainty if the calculator was actually used or not in each case. We started to ask providers to document use of the calculator in the discharge summary so we could better track that the changes observed between pre- and post-intervention cohorts were attributed to our intervention. We updated the discharge summary template to prompt the writer to document what prescription size the calculator recommended and what was prescribed.

We compared 95 pre-intervention patients to 95 post-intervention patients, including their demographic and perioperative characteristics and post-discharge survey responses. These cohorts were comparable for all sociodemographic characteristics shown in Table 3 as well as frequency of an opioid listed on the preoperative home medication list. They underwent similar surgeries (Table 4) and had comparable postoperative courses including similar utilization of epidurals, TAP blocks, and non-opioid oral analgesics. There was no difference in postoperative admission to the intensive care unit, use of antibiotics or red blood cell transfusions. Median length of stay was the same (4 days) for both cohorts. Rates of emergency room (ER) visits and readmission to a hospital within 30 days after surgery were no different in the pre- and post-intervention cohorts.

**Table 3:** Demographic characteristics of the pre-intervention versus post-intervention cohorts.

**Variable**	**Pre-intervention (n = 95)**	**Post-intervention (n = 95)**	**P value**
Age (years)	56 (43, 65)	59 (44, 68)	0.37
BMI (kg/m^2)	25.6 (22.9, 30.8)	26.2 (22.7, 31.9)	0.67
Race as per the EMR			0.68
White or Caucasian	54.7 %	47.4 %	
Black or African American	4.2 %	6.3 %	
Asian	18.9 %	24.2 %	
Other	22.1 %	22.1 %	
Hispanic ethnicity as per the EMR	16 %	19 %	0.54
Primary/preferred language as per the EMR			0.065
English	87.4 %	81.1 %	
Spanish	4.2 %	8.4 %	
Chinese (Cantonese and Mandarin)	5.3 %	1.1 %	
Other	3.2 %	9.5 %	
Preoperative use of an opioid medication*	27.4 %	18.9 %	0.17

Results are presented as median values (with interquartile range) or percentages, as indicated. EMR = Electronic medical record, BMI = Body mass index. *If listed on the home medication list prior to surgery.

**Table 4:** Perioperative outcomes of the pre-intervention versus post-intervention cohorts.

**Variable**	**Pre-intervention (n = 95)**	**Post-intervention (n = 95)**	**P value**
**Surgical procedure**			
Hysterectomy	66 %	77 %	0.11
Bowel resection (1 + )	16 %	22 %	0.27
Adnexal surgery	85 %	88 %	0.52
**Postoperative care**			
Length of stay (days)	4 (3, 5)	4 (3, 5)	0.99
Epidural use	44 %	43 %	0.82
TAP block use	49 %	52 %	0.77
Acetaminophen use	98 %	100 %	0.16
Gabapentin use	95 %	94 %	0.76
NSAID use	83 %	82 %	0.85
OME used the day before hospital discharge	18.6 (0, 45)	7.5 (0, 30)	0.08
**Postoperative complications**			
ICU admissions	7 %	9 %	0.77
Transfused red blood cells (units)	0 (0, 1)	0 (0, 1)	0.19
Antibiotic use	15 %	13 %	0.67
ER visit within 30 days post-surgery	3 %	7 %	0.22
Readmission within 30 days post-surgery	1 %	1 %	0.93

Results are presented as median values (with interquartile range) or percentages, as indicated.

Abbreviations: TAP = Transversus abdominis plane, NSAID = non-steroidal anti-inflammatory drug, OME = Oral morphine equivalents, ICU = Intensive care unit.

*If listed on the home medication list prior to surgery.

**Fig. 1:**
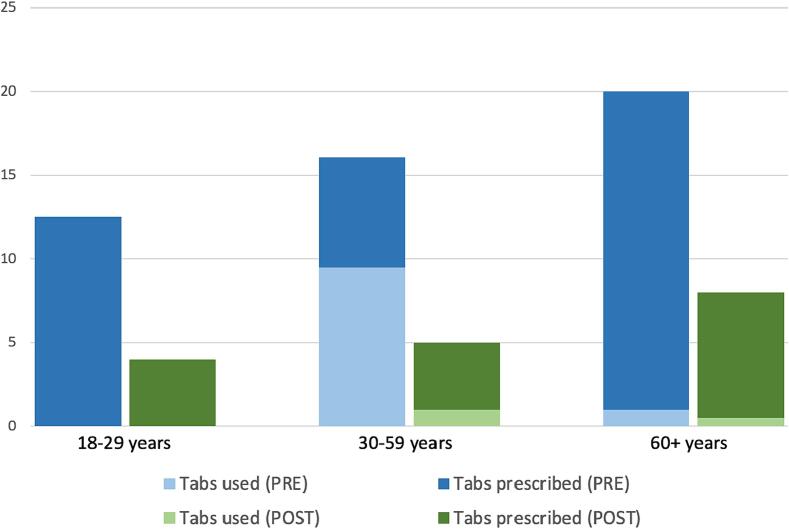
Outcomes of implementing our opioid prescription size calculator, comparing pre-intervention to post-intervention cohorts by age group. Median doses of 5 mg oxycodone tabs are displayed.

Following implementation of our calculator there was a significant decrease in the median opioid prescription size from the equivalent of 20 doses of 5 mg oxycodone to 5 doses (p < 0.01) (Fig. 1). Self-reported use of opioids at home decreased from 3 doses of 5 mg oxycodone to 1 dose, but was not a significant difference (p = 0.05). The refill rate remained the same and was 12.6 % pre-intervention and 11.6 % post-intervention (p = 0.82). Two patients pre-intervention and 2 patients post-intervention declined to fill their post-discharge prescriptions which were the equivalent of 5 and 20 doses of 5 mg oxycodone and 2 and 10 doses of 5 mg oxycodone, respectively. These four patients did not use any opioids at home. There was no difference in the percentage of patients who did not use any opioids at home (37 % pre-intervention and 49 % post-intervention, p = 0.08).

The surplus of leftover, unused opioids our patients reported having at home from initial post-discharge prescriptions (filled and unfilled) and refills decreased from 1264 doses of 5 mg oxycodone in the pre-intervention cohort, to 490 doses in the post-intervention cohort. This was a 61 % reduction in the quantity of leftover pills. The median amount of unused opioids ranged from a median of 14.2 doses of 5 mg oxycodone per patient pre-intervention (interquartile range 5, 20) to 5 doses of 5 mg oxycodone per patient post-intervention (interquartile range 2, 9.5). The patients’ reflections on their prescription size changed from pre- to post-intervention (p < 0.05) with more patients saying they received “too few” opioids (12.6 % vs 21.0 %), more patients reporting that the prescription size was “just right” (42.1 % vs 58.9 %), and fewer patients sharing that they had received “too many” opioid doses (45.3 % vs 20.0 %).

**Fig. 2:**
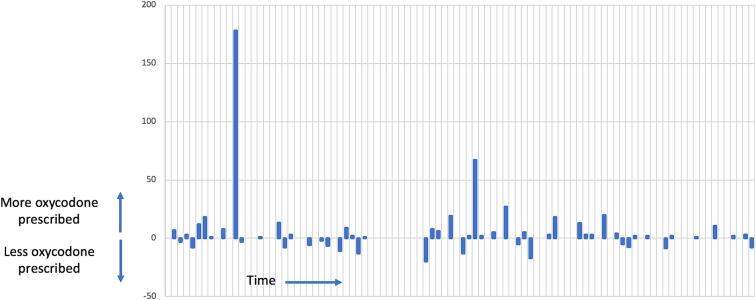
The difference in the number of 5 mg oxycodone doses prescribed compared to the calculator’s recommended prescription size for the post-intervention cohort.

Thirty-six patients (37.9 %) in the post-intervention cohort received a larger prescription than the calculator’s recommended. Adherence to the calculator’s recommended discharge prescription size improved over the course of the eight months of follow-up: the median amount of OME prescribed above the calculator’s recommended value was 52.5 OME in the first four months after the calculator was launched and decreased to 30 OME in the next four months. Starting in November 2022, 88.3 % of discharge summaries reported the recommended prescription size computed by the calculator and what quantity of opioids was actually prescribed. The amount of over-prescription above the calculator’s recommended prescription size ranged from the equivalent of 1–178 doses of 5 mg oxycodone (Fig. 2) despite these patients using a median of 1 dose of 5 mg oxycodone the day before hospital discharge (interquartile range 0, 3.5) and a median of 2.5 doses of oxycodone at home (interquartile range 0, 15). The only two patients who met criteria for opioid tolerance per FDA guidelines were over-prescribed the most (67 and 178 doses of 5 mg oxycodone above the calculators recommendation). Perfect use of the calculator would have led to a refill rate of 23.2 % based on post-intervention patients’ self-reported use of opioids at home. If the three (3.2 %) post-intervention patients who used > 40 doses of 5 mg oxycodone are excluded (the “outliers” that our model originally excluded when it was developed), perfect use of the calculator would have led to a refill rate of 20 %, which is 5 % greater than our target refill rate (15 %).

## 4 Discussion

In this quality improvement project, we successfully developed an easy-to-use post-discharge opioid prescription size calculator for patients after laparotomy using readily available objective data from the electronic medical record. We implemented this intervention on our inpatient service and successfully used it to decrease the median opioid prescription size for home by 75 % without increasing our refill rate. The primary aim of the project was to decrease the quantity of leftover, unused opioids we introduced into our patients’ communities and we saw a 61 % reduction in this data point.

We are not the first to approach the dilemma of postoperative opioid prescribing. This has evolved from uniform prescribing recommendations for an amount of opioids based on the surgical procedure done, to prescribing opioids based on inpatient hospital use of pain medications, to calculators like our own which utilize multiple patient factors to more precisely predict a patient’s need for opioids at home (Boitano et al., 2020, Glaser et al., 2020, Michigan Opioid Prescribing Engagement Network, 2020, Overton et al., 2018, Rodriguez et al., 2022, Straubhar et al., 2021, Straubhar et al., 2023). Similarly developed calculators have been published by groups from Duke University and the University of Michigan (Rodriguez et al., 2022, Straubhar et al., 2021, Straubhar et al., 2023). Their approaches combine minimally invasive and open surgical patients while we chose to focus on open surgical patients alone as our practice is to give no more than 5 doses of opioids after laparoscopic surgery. Our outcomes are comparable to both groups with respect to provider compliance rate, percent of patients not using any opioids after discharge to home, and refill rate.

A significant strength of our model is its simplicity. It only needs the input of two variables that are readily available in the EMR and does not require collection of additional data from patients. Additionally it utilizes only objective data and could be employed across surgical specialties. Multiple studies across clinical settings have demonstrated racial disparities in pain management and opioid prescribing (Kang et al., 2022, Keister et al., 2021, Morden et al., 2021, Romanelli et al., 2023). We intentionally chose not to use patient race/ethnicity or variables associated with socioeconomic status (e.g. the use of tobacco or alcohol or educational status) in our model to avoid including variables in our clinical algorithm that would perpetuate disparities rather than reducing them (Tong, 2021). Our model does not seek to explain why one patient would use more opioids than another or penalize someone who needs more opioids than their peers.

We acknowledge the challenge of definitively saying that the reductions we saw in opioid prescribing are due to our intervention. Prescription sizes in our division for patients after open surgery were already declining over the last decade. While not statistically significant, patients used less OME the day before discharge (18.6 pre- vs 7.5 post-intervention), implying that postoperative pain management in our institution has improved over this timeframe and/or that patients are increasingly eager to avoid opioids. It should also be noted that discharging providers still frequently prescribed larger amounts of opioids to the post-intervention cohort than what was recommended by the calculator, although 80 % of post-intervention prescriptions were at most 3 doses of 5 mg oxycodone above the recommended size or smaller. We argue that the magnitude and timescale of differences in opioids prescribed for home and reduction in unused opioids make it unlikely that the outcomes we saw were due to an existing trend of decreased prescription size. Additionally we saw a further decrease in over-prescription rates over the course of our intervention. We did not conduct a thorough review of each instance of over-prescription, but we hypothesize that reasons for over-prescription were the prescriber not reviewing the patient’s opioid use the day prior to discharge, potentially prepping the discharge prescriptions in advance and then not updating the opioid quantity when the patient actually left the hospital, or potentially feeling uncomfortable prescribing less than what we have historically done.

It is unknown whether strict adherence to the calculator’s recommendations would impact refill rates or patient satisfaction, particularly given the association between amount of opioids prescribed and amount of opioids used (Gomes et al., 2011, Howard et al., 2018). We took steps to enhance uptake of the calculator’s recommendations (i.e. educational interventions with residents rotating on the inpatient service and requesting documentation of the recommended prescription size in discharge summaries), however an emphasis of the calculator is that it is a recommendation, not a required prescription size. We recognize some patients and providers may feel more comfortable with a different prescription size. We support the findings of our paper as a representation of a real-life application of our tool with highly successful reduction in prescribed opioids and amount of leftover, unused opioids.

One limitation of our calculator is that it was based on a small sample size of patients with an age distribution skewed towards an elderly population. Additionally, 9 % of the patients from the pre-intervention cohort were excluded as outliers in the development of the calculator for using > 40 doses of 5 mg oxycodone at home. These patients were more likely to use opioids chronically, have a diagnosis of anxiety, and use > 37.5 OME on the day before discharge. The question of how many opioids to prescribe a patient with baseline chronic opioid use remains unanswered. Only three (3.2 %) patients in the post-intervention cohort used > 40 doses of 5 mg oxycodone.

As we commit to opioid stewardship and seek ways to prevent persistent opioid use for both our patients and their communities, we must continue to develop and use evidence-based tools that improve the precision and personalization of the post-surgical care that we provide. This opioid calculator was highly successful in our gynecologic oncology open surgical patient population in reducing the amount of unused opioids introduced into our patients’ communities. It is easy to use and was developed to avoid the introduction of implicit bias in our opioid prescribing. We are currently working with our information technology team to incorporate the calculator directly into our electronic medical record and seeking collaboration with other surgical specialties to apply this calculator.

## CRediT authorship contribution statement

**Allison H. Kay:** Writing – original draft, Visualization, Project administration, Methodology, Investigation, Formal analysis, Data curation, Conceptualization. **Rachel Levy:** Writing – review & editing, Project administration, Methodology, Investigation, Data curation. **Nancy Hills:** Writing – review & editing, Methodology, Investigation, Formal analysis. **Allyson Jang:** Writing – review & editing, Project administration, Investigation, Data curation. **Alison Mcgough-Maduena:** Writing – review & editing, Project administration, Investigation, Conceptualization. **Natalia Dematteo:** Writing – review & editing, Methodology, Investigation, Data curation. **Melissa Mark:** Writing – review & editing, Methodology, Investigation, Data curation. **Stefanie Ueda:** Writing – review & editing, Supervision, Project administration, Methodology, Data curation, Conceptualization. **Lee-may Chen:** Writing – review & editing, Visualization, Supervision, Project administration, Methodology, Investigation, Conceptualization. **Jocelyn S. Chapman:** Writing – review & editing, Supervision, Methodology, Investigation, Formal analysis, Data curation, Conceptualization.

## Declaration of Competing Interest

The authors declare that they have no known competing financial interests or personal relationships that could have appeared to influence the work reported in this paper.
